# Symptom Burden and Profiles in Concussed Children with and without Prolonged Recovery

**DOI:** 10.3390/ijerph17010351

**Published:** 2020-01-04

**Authors:** Samantha Schilling, Adam Mansour, Lindsay Sullivan, Kele Ding, Thomas Pommering, Jingzhen Yang

**Affiliations:** 1 Center for Injury Research and Policy, The Abigail Wexner Research Institute at Nationwide Children’s Hospital, Columbus, OH 43205, USA; schilling.120@buckeyemail.osu.edu (S.S.); mansouradam1995@gmail.com (A.M.); ginger.yang@nationwidechildrens.org (J.Y.); 2 Department of Health Sciences, Kent State University, Kent, OH 44240, USA; kding@kent.edu; 3 Department of Pediatrics, College of Medicine, The Ohio State University, Columbus, OH 43210, USA; thomas.pommering@nationwidechildrens.org; 4 Division of Sports Medicine, Nationwide Children’s Hospital, Columbus, OH 43205, USA

**Keywords:** concussion, pediatric, recovery, retrospective case-control study, symptom burden, symptom profile

## Abstract

Although symptom burden and symptom profile severity are independent predictors of post-concussion symptom duration, few studies have examined their effects on prolonged recovery simultaneously. This study examined differences in symptom burden and symptom profile scores between concussed children with prolonged recovery and those with typical recovery. We conducted a retrospective case-control study of concussed children aged 10–18 years. Prolonged recovery was defined as symptom duration beyond 28 days post-injury. Symptom burden was measured as total symptom score (TSS) at injury. Symptom profiles included: (1) vestibular, (2) ocular, (3) cognitive/fatigue, (4) migraine, and (5) anxiety. A total of 4380 unique concussions sustained by 3777 patients were included; 80.3% white, 60.0% male, and 44.0% aged 13–15 years. The prolonged recovery group had a significantly higher TSS and greater number of symptoms than the typical recovery group (*p* < 0.001 and *p* < 0.001, respectively). The prolonged recovery group had significantly higher scores on all five symptom profiles, including vestibular (*p* < 0.001), ocular (*p* < 0.001), cognitive/fatigue (*p* < 0.001), migraine (*p* < 0.001) and anxiety (*p* < 0.001), than the typical recovery group, even after adjusting for number of symptoms and other covariates. Further studies using prospective cohort designs are needed to better understand the influence of symptom burden and profiles on pediatric concussion recovery.

## 1. Introduction

Concussions are a growing public health concern, especially among children and adolescents as their brains are still developing [1,2,3]. It is estimated that, each year, up to two million sport- and recreation-related concussions occur among US children ≤18 years old [1]. Although most children recover within four weeks, up to 30% of children experience ongoing and persistent post-concussion symptoms (PPCSs), which could last weeks, months, or years post-injury [4,5]. Children with PPCSs often face adverse outcomes such as increased risk for poor academic performance [6,7], missed school days and social activities [6,7], and an overall decreased quality of life compared to children without PPCSs [8]. This underscores the importance of identifying predictors of prolonged concussion recovery in the pediatric population. Such knowledge may help clinicians identify at-risk children earlier and thus implement targeted concussion management and treatment plans.

Previous research has shown that demographic and injury characteristics such as adolescent age [4,9,10,11], female sex [9,10,12,13,14], history of prior concussion [15,16], and amnesia [5,12,17] are associated with prolonged recovery from concussion. Additional research shows that symptom burden, defined by a patient’s total symptom score (TSS), is the most consistent predictor of prolonged recovery among concussed youth [5,18,19,20,21,22]. Meehan and colleagues [21] found that a TSS ≥13 at time of injury was associated with an increased risk of prolonged symptoms among patients aged 7 years to 26 years [21]. However, symptom burden alone does not provide detailed information about the specific type of symptoms patients experience post-concussion.

Emerging evidence shows that the type of symptoms experienced post-concussion influences symptom duration [4,12,19,20,23,24]. Recent studies suggest that concussion symptom profiles, commonly referred to as concussion symptom factors or symptom clusters, are associated with symptom duration [25,26]. Concussion symptom profiles are used to group similar individual post-concussion symptoms into clinical subtypes [25,27,28,29,30]. The use of concussion symptom profiles allows clinicians to provide patients with more targeted treatment and therapies [19,20,25,27,28,29,30,31]. For example, concussed patients who present predominately with vestibular dysfunction, including motion/environmental sensitivity, dizziness, lightheadedness, imbalance, vertigo, nausea, and mental fogginess, may benefit from different treatment plans than concussed patients who present with anxiety/mood symptoms [27]. Symptom profiles are increasingly being used by clinicians, in addition to symptom burden, to help identify patients at risk for prolonged recovery and to address patient heterogeneity [25,26,27,28,29,30,31,32]. Prior studies report conflicting findings. Some studies found that symptom profiles that include headache, dizziness, fatigue, and fogginess are often associated with prolonged recovery and increased symptom duration among concussed youth [12,23] while others found that vestibular [27], emotionally-derived [12] and somatic symptoms [19,33] are associated with prolonged concussion recovery in children and adolescents.

To date, very few studies have examined the influence of symptom burden in combination with symptom profiles, two independent predictors of post-concussion symptom duration, on the risk of prolonged recovery among concussed youth [26]. To address this gap in the literature, the present study aimed to examine differences in symptom burden and profile scores among concussed children aged 10–18 with a prolonged recovery as compared to their counterparts with a typical recovery. We hypothesized that compared to the typical recovery group, the prolonged recovery group would be associated with significantly (i) higher symptom burden scores and (ii) greater vestibular, ocular, cognitive/fatigue, migraine, and anxiety symptom severities. The ability to better identify symptom profiles associated with prolonged recovery can inform clinical decision making and concussion management, allowing clinicians to provide early, targeted treatment.

## 2. Materials and Methods

### 2.1. Study Design

We conducted a retrospective case-control study of pediatric patients with concussion aged 10–18 years, with cases defined as concussions with a prolonged recovery and controls defined as concussions with a typical recovery.

### 2.2. Study Population

A total of 4951 physician-diagnosed concussions, among 4248 individual patients who were injured between 1 January 2012 and 31 December 2017, sought care at a large Midwest children’s hospital, and had visited one of seven affiliated concussion clinics at least once, were initially assembled for this study. Among these, 569 concussions were excluded due to a missing symptom score (*n* = 16), or a missing symptom resolution date (*n* = 553). A final cohort comprising 4380 unique concussions sustained by 3777 individual patients was included for the analyses. This study was approved by the Institutional Review Board at Nationwide Children’s Hospital (IRB17-00373).

All data for this study were obtained through electronic medical records, collected as part of routine clinical care by sports medicine physicians or athletic trainers. Concussion diagnoses were identified according to the International Classification of Diseases, 9th and 10th revisions (ICD codes 9 and 10, respectively). Inclusion criteria were as follows: youth aged 10–18 years; injuries occurring from 1 January 2012 to 31 December 2017; presentation to one of seven concussion clinics affiliated with our institution; and physician-confirmed acute signs or symptoms consistent with concussion. Exclusion criteria were defined as follows: patients who required neurosurgery or other surgical intervention following their concussion and patients who presented with intracranial hemorrhage.

### 2.3. Study Variables

Case definition: a prolonged recovery, an independent variable, was measured by length of clinician-confirmed symptom duration. Symptom duration was defined as the number of days from injury until symptom resolution (i.e., symptom free or symptom score less than 3). Symptom resolution was established through the 22 item Post-Concussion Symptom Scale (PCSS) (see Supplementary Table S1) [12,21,34] completed at the patient’s final clinical visit. We operationalized a prolonged recovery as a post-concussion symptom score of 3 or higher on or beyond 28 days post-injury; and a typical recovery, defined as acute symptoms resolved within 28 days post-injury or a post-concussion symptom score of zero or less than 3 by 28 days post-injury.

Symptom burden, one of the dependent variables, was measured using the PCSS [19,21,23,35]. As part of the standard concussion clinic evaluation, patients completed the PCSS at their initial clinic visit, including retrospectively ranking the severity of their symptoms at time of injury on a 7-point scale ranging from 0 (asymptomatic) to 6 (most severe). Symptom burden score at time of injury was calculated by summing the responses to the 22 items (possible range = 0–132). Reliability and validity of the PCSS is well established [34].

Symptom profiles, another dependent variable, was derived from the 22 item PCSS by clustering the symptoms into five symptom profiles. Based on a previously published study [27], patients’ symptoms were classified into the following five symptom profiles based on their post-concussion symptom score at injury: (1) vestibular (possible range = 0–24), (2) ocular (possible range = 0–24), (3) cognitive/fatigue (possible range = 0–30), (4) migraine (possible range = 0–6), and (5) anxiety (possible range = 0–24) (Table 1).

Other variables included age, sex, race, date of injury, history of prior concussion, history of learning disorder(s), loss of consciousness, retrograde amnesia, anterograde amnesia, and neck pain.

### 2.4. Statistical Analysis

Data analyses were conducted using SAS software, version 9.4 (SAS Institute Inc., Cary, NC, USA). Descriptive statistics were used to describe patient and injury characteristics along with recovery patterns. Chi-squared tests were used to compare the frequency distributions of patient and injury characteristics between patients with prolonged recovery versus those with typical recovery. Independent *t*-tests were conducted to compare differences in total symptom burden score and four of the five symptom profile scores at time of injury between patients with and without prolonged recovery. Due to the skewed distribution of the anxiety symptom profile, the Kruskal–Wallis test was used to compare the difference in median between the prolonged and typical recovery groups. Multiple regression analyses were conducted to examine differences in symptom profile severity scores between the prolonged recovery and typical recovery groups, adjusting for patient characteristics (e.g., sex, age), total number of symptoms at injury, variables showing statistical significance in the binary analysis (e.g., history of prior concussion, history of learning disorder, retrograde amnesia, anterograde amnesia, and neck pain), and other symptom profiles. Due to the collinearity caused by the high correlations between symptom profiles, we first regressed each symptom profile of interest against the other four symptom profiles and then included and adjusted the residual in the multiple regression model while examining differences in the symptom profile severity score between the prolonged and typical recovery groups [36]. Additionally, a log transformation was performed on the anxiety symptom profile to deal with the skewed distribution of the variable and to stabilize the variability of residuals from the regression models. In all cases statistical significance was determined by an α level of *p* < 0.05.

## 3. Results

### 3.1. Patient and Injury Characteristics

A total of 4380 concussions sustained by 3777 patients were included, with predominantly white (80.3%), male (60.0%), and patients aged 13–15 years old (44.0%) (Table 2). Over one-third of concussions (35.0%) had a prior concussion history. More than half of concussions (62.9%) were accompanied by neck pain at time of injury. A total of 570 (13.5%) concussions experienced loss of consciousness at time of injury (Table 2). Nearly two thirds of concussions (63.5%) initially presented to the concussion clinic within 10 days of injury, while 8.5% of concussions presented on or after day 29 post-injury. The median length of symptom duration was 19.5 days.

Of the 4380 unique concussions, 1612 (36.8%) experienced prolonged recovery. Compared to concussed children with a typical recovery, children with a prolonged recovery were more likely to be female (*p* < 0.001), be in the older age group (*p <* 0.01), have a history of prior concussion (*p* < 0.01), and have a history of a learning disorder (*p* < 0.001). The prolonged recovery group was also more likely to experience retrograde amnesia (*p* < 0.001), anterograde amnesia (*p* < 0.001), or neck pain (*p* < 0.001) at time of injury than the typical recovery group (Table 2).

### 3.2. Differences in Total Symptom Score and Each Symptom Profile Scores by Recovery Group

Average TSS was 35.8 at injury (Median = 33.0). Average number of symptoms experienced at injury was 13.0 symptoms (Median = 13.0) (Table 3).

The prolonged recovery group had a significantly higher TSS and greater number of symptoms at injury than the typical recovery group (*p* < 0.001 and *p* < 0.001, respectively) (Table 3). The prolonged recovery group also had significantly greater vestibular (*p* < 0.001), ocular (*p* < 0.001), cognitive/fatigue (*p* < 0.001), migraine (*p* < 0.001), and anxiety (*p* < 0.001) symptom profile severity scores than the typical recovery group (Table 3). A higher severity score on any individual symptom profile was significantly associated with elevated severity scores on all remaining symptom profiles (*p* < 0.001). The pearson correlation coefficients ranged from r = 0.80 (*p* < 0.001) for the correlation between the cognitive/fatigue and vestibular symptom profiles to r = 0.31 (*p* < 0.001) for the correlation between the migraine and anxiety symptom profiles.

### 3.3. Adjusted Analysis on Symptom Profiles by Recovery Group

Unadjusted analyses revealed that patients experiencing prolonged symptoms were significantly more likely to have higher severity scores on all five symptom profiles than the typical recovery group (Table 4). After adjusting for covariates, the prolonged recovery group had significantly higher vestibular (β = 0.49; 95% CI: 0.33, 0.65; *p* < 0.001), ocular (β = 0.50; 95% CI: 0.30, 0.70; *p* < 0.001), cognitive/fatigue (β = 0.78; 95% CI: 0.53, 1.03; *p* < 0.001), migraine (β = 0.08; 95% CI: 0.03, 0.13; *p* < 0.001) and anxiety (β = 0.08; 95% CI: 0.05, 0.10; *p* < 0.001) symptom profile severity scores at time of injury as compared to the typical recovery group. Greater number of symptoms experienced at injury were associated with higher symptom severity scores on all five symptom profiles.

Older age was associated with lower severity scores on the migraine symptom profile (*p* < 0.05), while female sex was significantly associated with a greater ocular (*p* < 0.001), migraine (*p* < 0.001) and anxiety (*p* < 0.001) symptom severity score (Table 4). Both retrograde and anterograde amnesia at injury were associated with increased severity scores on all symptom profiles (*p* < 0.001), with the exception of the relationship between anterograde amnesia and the migraine symptom profile. Neck pain at injury was associated with greater scores for all symptom profiles (*p* < 0.05) except cognitive/fatigue (Table 4).

## 4. Discussion

This study examined differences in symptom burden and symptom profile scores between concussed children aged 10–18 who had a prolonged recovery and those who had a typical recovery. The main findings show that the prolonged recovery group had significantly higher symptom burden at injury than the typical recovery group; and that the prolonged recovery group also had higher severity scores on all five symptom profiles (vestibular, ocular, cognitive/fatigue, migraine, and anxiety) at injury than the typical recovery group after adjusting for symptom burden and other covariates. These findings add to the current literature on the relationship between symptom profiles and prolonged recovery in pediatric concussion patients, although prospective studies are needed to confirm these findings. Understanding symptom profiles could help inform a targeted approach to more effective care of children following concussion.

Consistent with previous studies [5,19,20,21,22], we found that the prolonged recovery group had an increased symptom burden, including TSS and number of symptoms, at injury. Meehan et al. [21] found that overall symptom burden is the only independent predictor of prolonged symptoms following sport-related concussion [21]. Our findings are also supported by research conducted by Heyer and colleagues [12] which found that either TSS or specific symptom profiles were a significant predictor of recovery duration. Our results confirm the importance of prospectively examining symptom burden at injury to identify patients at risk of prolonged recovery and to develop effective, tailored concussion treatment plans based on the patients’ specific symptom presentation.

We found that the prolonged recovery group had higher severity scores on all five symptom profiles (vestibular, ocular, cognitive/fatigue, migraine, and anxiety), when adjusting for patient characteristics, symptom burden, as well as other covariates. Our findings support recent studies that found that the vestibular [27,37], ocular [27,38], and migraine [24,27,39] symptom profiles are associated with an increased risk of prolonged recovery, and that emotional and cognitive/fatigue symptom profiles at initial clinic presentation are associated with prolonged symptom duration [12]. Our findings, in line with others [12,23,27,40], suggest that symptom profile severity scores at injury are clinically relevant risk factors associated with concussion symptom duration among children and adolescents. More prospective studies could help further our understanding of how symptom profiles are associated with prolonged concussion recovery.

Our results showed that a higher severity score on any individual symptom profile was significantly associated with elevated severity scores on all remaining symptom profiles. For example, a high severity score on the cognitive/fatigue symptom profile was associated with higher severity scores on the vestibular, ocular, migraine, and anxiety symptom profiles. This finding is consistent with prior research [27], which suggests that there is a complex interaction between symptom profiles. Possibly, post-concussion symptoms may cross-load into more than one symptom profile; thus, the symptoms used in this study to classify symptom profiles may be partially responsible for this interaction. Additionally, as suggested by others [25,27], concussion patients may present with more than one symptom profile. Clinicians should consider these relationships between symptom profiles when assessing and determining treatment plans for concussed youth.

In recent years, knowledge on symptom profile classification systems has grown rapidly [19,20,25,27,28,29,30,31,40]. However, there is currently no standardized protocol for the clinical use of symptom profiles in concussion management, resulting in a wide variation in the number and types of symptom profiles analyzed, along with differences in the categorization of symptoms within their respective profiles across the literature. For the purposes of this study, we utilized the five-factor classification system established by Kontos and colleagues [27], which includes vestibular, ocular, cognitive/fatigue, migraine, and anxiety symptom profiles. The strengths of these symptom profiles, developed by Kontos et al. [27], include the ability to classify concussion symptoms into symptom profiles in the first week after injury and the symptom profiles are not mutually exclusive and thus a patient may have a primary, secondary, or tertiary symptom profile [27,29]. Additionally, each symptom profile has specific assessment and treatment plans [27,29]. Unlike other classification systems that use discrete cutoff values to categorize symptoms into symptom profiles [35], this classification system is limited by its inability to provide clinicians with a precise numerical value that can be factored into concussion diagnosis and/or treatment as a predictor of prolonged recovery. Although knowledge on symptom profiles is growing rapidly [19,20,25,27,28,29,30,31,40], no such symptom profile classification system has been empirically validated [29]. With a more unified approach to the classification of symptom profiles, clinicians could provide concussed patients with more individualized treatments based on their symptom profile severity scores, combined with their symptom burden at time of injury, and patient and injury characteristics.

Unlike previous research [40], we found that older age was associated with lower severity scores on the migraine symptom profile. Possibly, this difference in results could be related to differences in the symptom profile classification systems used or the populations studied. Kontos et al. [40] assessed age differences in symptom profiles, using a 4-factor classification system (cognitive-fatigue-migraine, affective, somatic, and sleep), between high school and college athletes following a sport-related concussion, while we examined age differences in youth aged 10–18 years following a sport- or non-sport-related concussion. Another potential explanation for this difference in results is that unlike prior studies our analyses adjusted for symptom burden. Further research is needed to confirm these results.

Our study, in line with others [40,41,42], found that female sex was significantly associated with increased ocular, migraine, and anxiety symptom severity scores. One potential explanation for this sex difference in anxiety symptom severity scores is that females are more likely to report experiencing emotional disturbances or symptoms than their male counterparts [42,43,44,45]. A potential reason for the observed sex difference in the migraine symptom severity scores, with the migraine symptom cluster including headache, is that the prevalence of migraine is higher in females than males [46]. This is supported by previous research [47,48]. Prospective studies are needed to identify patient and injury characteristics associated with each symptom profile among youth following both sport- and non-sport-related concussions.

### Limitations

This study has several limitations. First, the retrospective nature of this study did not allow us to draw a causal relationship on how symptom burden and symptom profiles affect the risk of prolonged recovery. Additional prospective studies are needed to confirm this study’s findings. Second, all patients in our study sought medical care at a concussion clinic. This may have resulted in the selection of a cohort with more severe concussions as compared to those who do not seek care at a specialty clinic or who do not seek any care; thus, the results of our study may not be generalizable to all pediatric concussion patients. This study also relied on the retrospective recall of post-concussion symptoms at injury, which may have resulted in reporting bias and the underreporting of symptom severity [49] or possibly introduced recall bias into the PCSS scoring. We defined the prolonged recovery group using the symptom score of 3 or higher on or beyond 28 days post-injury, without considering pre-injury condition(s) that may result in symptoms similar to post-concussion symptoms. Thus, our results could have overestimated the number of concussed children in the prolonged recovery group. Finally, to determine participant’s symptom profile, we used symptoms reported at time of injury. We did not use other clinical information, including medical history, risk factors and injury information, and clinical examination, to determine symptom profiles. Future studies should use a more comprehensive assessment of symptoms and impairment after concussion to determine one’s primary symptom profile.

## 5. Conclusions

Our findings revealed that concussed children aged 10–18 with prolonged recovery had a higher symptom burden and higher severity scores on the vestibular, ocular, cognitive/fatigue, migraine, and anxiety symptom profiles at time of injury than children with a typical recovery. These data provide clinicians with clinically important information useful in identifying patients that may be at increased risk for prolonged recovery. Additional prospective studies on the influence of symptom profiles on concussion recovery outcomes are warranted to further our understanding of the clinical relevance of symptom profiles and help identify risk factors for prolonged recovery among concussed youth. Such empirical evidence can also be used to guide clinicians to provide patients with a more individualized and targeted concussion management plan, including the need for academic accommodations, and can inform physicians’ decisions on when patients can safely return to learn or play post-injury.

## Figures and Tables

**Table 1:** Classification of Symptom Profiles *.

Vestibular	Ocular	Cognitive/Fatigue	Migraine	Anxiety
Dizziness Nausea Mentally foggy Balance problems	Sensitivity to light Visual problems Headache Difficulty concentrating	Difficulty remembering Feeling slowed down Mentally foggy Difficulty concentrating Fatigue	Headache	Sadness Nervousness Feeling more emotional Irritability

* Symptom cluster classification was based on the classification of Kontos et al. [27].

**Table 2:** Patient and Injury Characteristics by Prolonged and Typical Recovery Groups.

Characteristics	All *n* (%)	Prolonged Recovery *n* (%)	Typical Recovery *n* (%)	*p* *
Total	4380	1612 (36.8%)	2768 (63.2%)	
Sex				<0.001
Female	1754 (40.0)	827 (51.3)	927 (33.5)	
Male	2626 (60.0)	785 (48.7)	1841 (66.5)	
Age group, y				0.005
10–12	584 (13.3)	185 (11.5)	399 (14.4)	
13–15	1927 (44.0)	698 (43.3)	1229 (44.4)	
16–18	1869 (42.7)	729 (45.2)	1140 (41.2)	
Race				0.462
White	3519 (80.3)	1300 (80.6)	2219 (80.2)	
Black	481 (11.0)	166 (10.3)	315 (11.4)	
Other	380 (8.7)	146 (9.1)	234 (8.5)	
Prior concussion				0.005
0	2850 (65.1)	1023 (63.5)	1827 (66.0)	
1	1015 (23.2)	366 (22.7)	649 (23.4)	
≥2	515 (11.8)	223 (13.8)	292 (10.5)	
History of learning disorder				<0.001
No	3807 (86.9)	1355 (84.1)	2452 (88.6)	
Yes	573 (13.1)	257 (15.9)	316 (11.4)	
Loss of consciousness				0.374
No	3661 (86.5)	1336 (85.9)	2325 (86.9)	
Yes	570 (13.5)	219 (14.1)	351 (13.1)	
Retrograde amnesia				<0.001
No	3461 (81.3)	1201 (76.4)	2260 (84.2)	
Yes	796 (18.7)	372 (23.6)	424 (15.8)	
Anterograde amnesia				<0.001
No	3511 (82.6)	1231 (78.3)	2280 (85.0)	
Yes	742 (17.4)	341 (21.7)	401 (15.0)	
Neck pain				<0.001
No	1625 (37.1)	434 (26.9)	1191 (43.1)	
Yes	2752 (62.9)	1177 (73.1)	1575 (56.9)	

* *p*-values are based on chi-square tests.

**Table 3:** Symptom Burden and Symptom Profiles at Time of Injury by Recovery Group.

	Recovery Group			
	All (*n* = 4380) Mean (SD)	Prolonged Recovery (*n* = 1612) Mean (SD)	Typical Recovery (*n* = 2768) Mean (SD)	*p*
Symptom Burden				
Total symptom score	35.8 (22.1)	42.9 (23.1)	31.7 (20.4)	<0.001 ^a^
Number of symptoms	13.0 (4.8)	14.2 (4.6)	12.2 (4.8)	<0.001 ^a^
Symptom Profile Severity Scores				
Vestibular	7.1 (4.9)	8.4 (5.0)	6.4 (4.7)	<0.001 ^a^
Ocular	10.3 (5.4)	11.7 (5.5)	9.5 (4.7)	<0.001 ^a^
Cognitive/fatigue	10.6 (7.9)	13.0 (8.2)	9.3 (7.5)	<0.001 ^a^
Migraine	4.1 (1.5)	4.3 (1.5)	3.9 (1.5)	<0.001 ^a^
Anxiety	2 (0, 6) ^b^	4 (0, 9) ^b^	2 (0, 5) ^b^	<0.001 ^c^

^a^: *p*-values are based on independent *t*-tests, ^b^: Medians (P25, P75) are presented due to skewed distribution, ^c^: *p*-values are based on Kruskal–Wallis tests.

**Table 4:** Unadjusted and Adjusted Regression Analyses on Symptom Profiles by Recovery Group.

	Symptom Profile				
Variable	Vestibular	Ocular	Cognitive/Fatigue	Migraine	Anxiety ^c^
	β (95% CI)	β (95% CI)	β (95% CI)	β (95% CI)	β (95% CI)
Unadjusted analysis					
Prolonged vs. typical recovery	1.98 (1.69, 2.28) **	2.22 (1.90, 2.55) **	3.69 (3.21, 4.16) **	0.37 (0.28, 0.46) **	0.37 (0.29, 0.46) **
Adjusted analysis ^a,b^					
Prolonged vs. typical recovery	0.49 (0.33, 0.65) **	0.50 (0.30, 0.70) **	0.78 (0.53, 1.03) **	0.08 (0.03, 0.13) **	0.08 (0.05, 0.10) **
Age, y	0.01 (−0.03, 0.04)	−0.05 (−0.10, 0.00)	−0.02 (−0.08, 0.05)	−0.02 (−0.03, 0.00) *	0.00 (−0.01, 0.01)
Sex (female vs. male)	0.08 (−0.07, 0.23)	0.29 (0.09, 0.48) **	0.22 (−0.03, 0.47)	0.11 (0.06, 0.16) **	0.03 (0.01, 0.06) **
Number of symptoms	0.61 (0.60, 0.63) **	0.67 (0.65, 0.69) **	1.12 (1.09, 1.14) **	0.13 (0.13, 0.14) **	0.09 (0.88, 0.93) **
Prior concussion	−0.01 (−0.16, 0.15)	0.02 (−0.18, 0.21)	0.02 (−0.23, 0.27)	−0.02 (−0.07, 0.03)	0.00 (−0.03, 0.03)
History of learning disorder	0.04 (−0.17, 0.26)	0.03 (−0.25, 0.30)	0.22 (−0.12, 0.57)	0.01 (−0.06, 0.09)	0.02 (−0.01, 0.05)
Retrograde amnesia	1.04 (0.84, 1.24) **	1.02 (0.77, 1.27) **	1.43 (1.11, 1.75) **	0.11 (0.04, 0.17) **	0.14 (0.11, 0.17) **
Anterograde amnesia	0.82 (0.61, 1.02) **	0.66 (0.40, 0.92) **	1.10 (0.77, 1.43) **	0.03 (−0.04, 0.09)	0.10 (0.07, 0.13) **
Neck pain	0.19 (0.03, 0.35) *	0.36 (0.16, 0.57) **	0.23 (−0.02, 0.49)	0.06 (0.01, 0.11) *	0.05 (0.02, 0.07) **

Note. * *p* < 0.01; ** *p* < 0.001. ^a^: The analyses were adjusted for patient characteristics (e.g., sex, age), total number of symptoms at injury, variables showing statistical significance in the binary analysis (e.g., history of prior concussion, history of learning disorder, retrograde amnesia, anterograde amnesia, and neck pain), and other symptom profiles. ^b^: Due to the collinearity caused by the high correlations between symptom profiles, we first regressed each symptom profile of interest against the other four symptom profiles and then included and adjusted the residual in the multiple regression model when examining differences between the prolonged vs. typical recovery groups. ^c^: A log transformation was performed on the anxiety symptom profile to deal with the skewed distribution of the variable and to stabilize the variability of residuals from the regression models.

## Supplementary Materials

The following are available online at https://www.mdpi.com/1660-4601/17/1/351/s1, Table S1: Post-Concussive Symptom Scale.
