# Supplementary material for: Symptom Burden and Profiles in Concussed Children with and without Prolonged Recovery
Source: Int J Environ Res Public Health. 2020 Jan 4;17(1):351. doi: 10.3390/ijerph17010351 (PMC6981707; doi:10.3390/ijerph17010351)
Supplement: Supplementary file 1 [file ijerph-17-00351-s001.pdf]

Supplementary Information

Table S1. Post-Concussive Symptom Scale.

Symptom	None	Mild		Moderate		Severe	
	0	1	2	3	4	5	6
Headache	0	1	2	3	4	5	6
Numbness	0	1	2	3	4	5	6
Nausea	0	1	2	3	4	5	6
Vomiting	0	1	2	3	4	5	6
Balance Problems	0	1	2	3	4	5	6
Dizziness	0	1	2	3	4	5	6
Fatigue	0	1	2	3	4	5	6
Trouble falling asleep	0	1	2	3	4	5	6
Excessive sleep	0	1	2	3	4	5	6
Sleeping less than usual	0	1	2	3	4	5	6
Drowsiness	0	1	2	3	4	5	6
Sensitivity to light	0	1	2	3	4	5	6
Sensitivity to noise	0	1	2	3	4	5	6
Irritability	0	1	2	3	4	5	6
Sadness	0	1	2	3	4	5	6
Nervousness	0	1	2	3	4	5	6
Feeling more emotional	0	1	2	3	4	5	6
Mentally foggy	0	1	2	3	4	5	6
Feeling slowed down	0	1	2	3	4	5	6
Difficulty remembering	0	1	2	3	4	5	6
Difficulty concentrating	0	1	2	3	4	5	6
Vision problems	0	1	2	3	4	5	6

Directions: Use the scale to complete the following table. 0 =no symptoms to 6 = severest symptoms.
